# The value of ethics in health communication

**Published:** 2008-11-15

**Authors:** Popa Florian

**Affiliations:** * ”Carol Davila” University of Medicine and Pharmacy, Bucharest, Romania

**Fig. 1:**
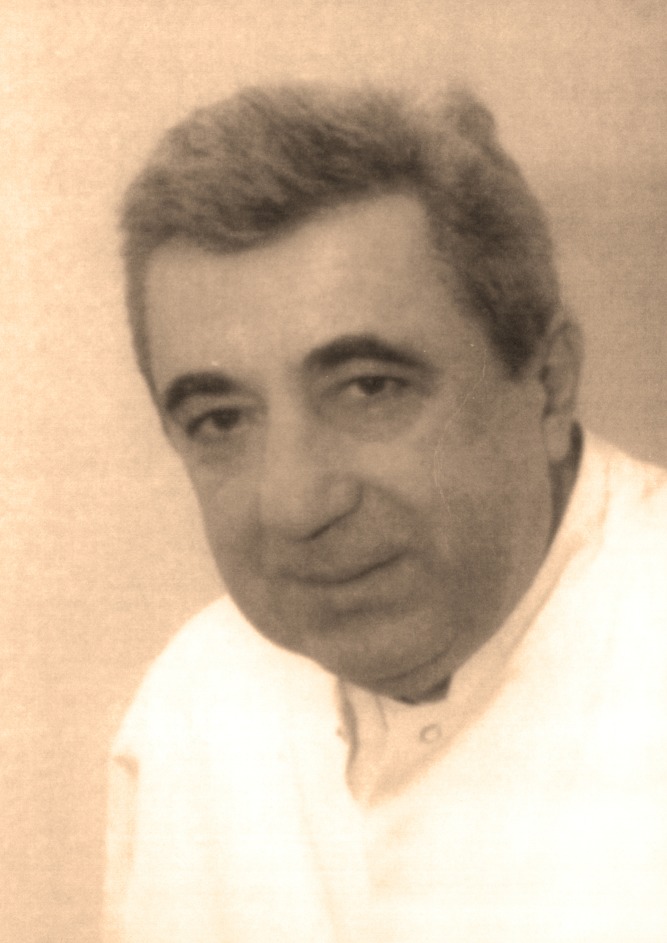
Opening ceremony of the Congress – Academician Ioanel Sinescu, Rector of “Carol Davila” University of Medicine and Pharmacy

Many health managers consider that their main duty consists in representing their institution’s interests and, by this, defending the patient’s and community’s welfare.

A manager should consider in all his decisions three ways in which the concept and rules of ethics can be valued and appreciated:

1. Keeping in mind the majority’s well-being

2. Defending the individual rights for all members of the institution

3. Ensuring the principles of equity, fairness, and objectivity

It is for sure that, in the real life, this challenge is hard to achieve if not impossible. Every manager knows to communicate inside and outside of its institution, in order to define its image as correctly as possible.

Medical institutions possess ethical values respected and appreciated by all its members. They always need to permanently reconsider their attitude related to ethical principles both inside the institution, and in all reciprocal actions with social environment.

The most important category of medical institutions is represented by hospitals. This type of institution is often confronted with at least two kinds of ethical issues:

1. On the one hand, there are issues regarding the important relations established between physician and patient during medical research activities, which had to be controlled through well-defined and clear rules and regulations (e.g. informed consent).

2. On the other hand, there are issues regarding internal relations, as well as relations with other institutions within or outside the system, for which no specific regulations are stated.

The main actors in every medical institution are: the specialized personnel, meaning individuals providing different medical services, including trainee and training personnel; the executive personnel without any medical background, but with economical, financial, marketing and management skills; and, last but not least, the patients with all their needs. All together form three groups, which ought to work together by communication based on the general and special ethical principles.

In these circumstances, a certain competition develops between medical and administrative personnel, which may be explained not only through their different background, but also through their different way of thinking.

The medical personnel considers as prevailing all values generated by the application of medical ethics, with special emphasis on the patients’ rights to be informed and to decide if they agree with the intended therapy, while the administrative personnel is always guided by marketing principles, as they target the best development of their institution.

Any deficiency in communication between these two important factors could generate real conflicts. Therefore, it is mandatory for different categories of personnel to communicate effectively in order to deliver a better quality of life for patients.

Another very important category of relations built inside a medical institution is communication with society. The way resources are used on short an on long terms, in order to ensure all the necessary services, could have also ethical implications on local community.

Developing a correct and honest method of communication between medical institutions is also very important for assuring specific kind of services for the community, without raise useless and false competitions.

One of the major responsibilities of the manager is to assure a real collaboration between all members of the institution, and so the ethical values could be valued in medical practice. In this way, it is highly recommended and useful to generate documents regarding ethical principles which have to guide medical and research activities: one document should statue current and future tasks of the institution, completed by an ethical code defining for each institution their own values and ethical principles guiding their activities.

In spite of the fact there are different such codes for each professional group, there has to be a common point of view for the institution which sets the responsibilities generated by joint activities and interpersonal relations.

Ethical issues regarding communication arise not only within the institution, but also from the outside of it. For example, the moral obligation of the manager to admit and to decide whether or not to make public medical errors which may occur.

As time passing by, a certain degree of reticence has been noticed admitting frankly “what went wrong” due to a fear which could be justified by possible legal and financial consequences. Therefore, in order to assure an appropriate quality of medical services in every day practice, there should be a permanent concern regarding compliance with ethical regulations. This is an essential fact not because it is fair but also because it provides a real support in resisting the high pressure which all medical suppliers have to face as a consequence of social and economical conditions. This way, the confidence which should be granted to medical staff can be encouraged.

In this context, it is mandatory for every manager belonging to the health system to consider that the attention paid to the integration of ethical principles of communication in daily activity does not mean a breach in prior practices and behaviors but a logical evolution to a broader approach which focus on all the institutions’ actions and participates in the process of decision making and manager – medical staff – patient communication.

Some of the medical institutions’ managers have a rigid attitude in admitting the errors happened while giving medical or surgical care to patients, considering that it is their duty to protect their institutions from all undesired legal obligations. Such attitude, besides its’ legal and financial consequences, has also important ethical implications.

Those who are against an open recognition of their own errors, have as a single argument the fact that patients take legal actions only if they are aware of medical errors. Therefore, one can consider that acknowledging the errors only increases the risk of legal actions taken against the institution.

On the other hand, it has been scientifically proven that in the case of errors with minor or moderate consequences, the majority of patients (up to 88%) do not have the intention to take legal actions if mistakes are recognized. This is more kept up if the honest acknowledgement allows compensatory measures. Meanwhile, the number of those who intend to take legal actions is growing significantly if the occurred errors are concealed by specialized personnel and discovered by the patient. Furthermore, the risk of losing credibility is arising.

That is why in the actual economical and social context the manager’s role becomes more complex in approaching and applying the ethical principles in communication.

